# Association of ABCB1 Gene Polymorphisms and Clopidogrel Responsiveness in Iranian Patients undergoing Percutaneous Coronary Intervention

**DOI:** 10.22037/ijpr.2020.1101083

**Published:** 2020

**Authors:** Soha Namazi, Ebrahim Sahebi, Negar Azarpira, Javad Rostami-Yalmeh, Javad Kojuri, Andia Khalili

**Affiliations:** a *Department of Clinical Pharmacy, School of Pharmacy, Tehran University of Medical Sciences, Tehran, Iran. *; b *Department of Pharmacotherapy, School of Pharmacy, Shiraz University of Medical Sciences, Shiraz, Iran. *; c *Transplant Research Center, Shiraz University of Medical Sciences, Shiraz, Iran. *; d *Department of Cardiology, School of Medicine, Shiraz University of Medical Sciences, Shiraz, Iran.*

**Keywords:** Clopidogrel, ABCB1 polymorphism, P-glycoprotein, Responsiveness, Percutaneous coronary intervention

## Abstract

Clopidogrel is an antiplatelet agent currently used for preventing stent thrombosis. Despite certain clinical benefits of clopidogrel in patients undergoing percutaneous coronary intervention (PCI), adequate antiplatelet effect has not been obtained in some patients. The present study was designed to investigate the potential association of ABCB1 (ATP-Binding Cassette, Subfamily B, member1) gene polymorphism, and clopidogrel responsiveness in Iranian patients after PCI. Sixty-seven patients were included in the study. Blood samples were taken from patients at baseline, 2 h after administration of 600-mg loading dose of clopidogrel, 24 h and 30 days after PCI. Platelet aggregation was measured by light transmittance aggregometry (LTA) with two levels of adenosine diphosphate (ADP) concentrations (5 and 20 µM). ABCB1 genotyping was performed by restriction fragment length polymorphism-polymerase chain reaction (RFLP-PCR). The allelic frequencies of wild type, heterozygote, and homozygote genotypes of ABCB1 were 20.9%, 74.6%, and 4.5%, respectively. There was no significant association between polymorphism of ABCB1 and clopidogrel non-responsiveness (*P *> 0.05) in various situations. No significant difference was observed for demographic characteristics. Genetic and demographic factors had no significant effect on the platelet activity of clopidogrel in an Iranian population.

## Introduction

Clopidogrel is an oral antiplatelet drug used to inhibit ADP-mediated platelet activation and aggregation in coronary artery disease, peripheral vascular disease, and to prevent myocardial infarction. Clopidogrel, as a prodrug is absorbed in the intestine and subsequently activated in the liver as a prodrug. CYP2C19 and CYP3A4/5 isomers of cytochrome P450 (CYP450) are the major enzymes involved in the metabolism of clopidogrel (1). Administration of clopidogrel prevents thrombotic events through irreversible blocking of purinergic P2Y12 receptor on the platelet surface (2, 3).

In ischemic events after percutaneous coronary intervention (PCI), the effective platelet inhibition requires clopidogrel and aspirin as dual anti-platelet therapy (4, 5). Moreover, many studies demonstrated that inter-individual variability in anti-platelet effect of clopidogrel. Approximately, 4 to 30% of patients did not attain adequate responsiveness (3, 6-8). Although the certain mechanism of this variability has remained unclear yet, pharmacogenomic analysis revealed that genetic polymorphisms of pharmacokinetic characteristics such as intestinal absorption (Multi Drug Resistance 1 (MDR1) gene polymorphisms) and metabolic oxidation (CYP450 polymorphisms) might be involved in these poor responses (9-12). ABCB1 encodes the P-glycoprotein (P-gp), a member of ABC transporter family proteins, that plays a critical role in the absorption, distribution, metabolism, and excretion (ADME) processes of various kinds of drugs (13, 14). P-glycoprotein is highly expressed in the liver, colon, small intestine, kidney, and adrenal (15). Expression of intestinal P-gp on the apical membrane of mature epithelial cells leads to the restriction of the absorption of the orally administered drugs (16).

Our previous study in Iranian population revealed that CYP450 iso enzymes polymorphisms (2C19 and 3A4) are not associated with poor clopidogrel responsiveness (17). In comparison with several other studies that found the maximum platelet aggregation 2 h after 600 mg loading dose of clopidogrel, the most frequent non-responsiveness of clopidogrel was also observed within 2 h after clopidogrel administration in our study (18-22).

A number of studies implied that there is an association between clopidogrel absorption and ABCB1 polymorphisms (9, 10 and 23). Until now, more than 50 single nucleotide polymorphisms (SNPs) have been investigated for ABCB1 gene. Among them C1236T, C3435T and G2677T/A are more common which C3435T has been shown to restrict the absorption of clopidogrel (10, 24). Moreover, Nakamura *et al.* and Moriya *et al.* reports also revealed that the C3435T polymorphisms affect P-gp expression in duodenal enterocytes (25, 26).

Altogether, we presumed that the disruption of the absorption may contribute to this non-responsiveness phenomenon. In the present study, we investigated the association of ABCB1 gene polymorphisms (C3435T) and clopidogrel responsiveness in Iranian patients after PCI in Iranian patients.

## Experimental

*Patient population*

Sixty-seven patients, enrolled in this cross sectional study, were participants of our previous survey in Shiraz as referral treatment center of south of Iran (17). Ethic committee of Shiraz University of Medical Sciences approved our study and a written informed consent was obtained from all patients. The patients with history of acute myocardial infarction (AMI) within 1 week, any contraindication to aspirin and clopidogrel, thrombocytopenia (platelets < 100 × 103), anemia (hemoglobin < 10 g/dL), renal failure (serum creatinine > 2.5 mg/dL) and patients treated with the drugs that interfere with clopidogrel, CYP3A4, and CYP2C19 metabolism (*i.e*., abciximab, dipyridamol, warfarin, phenytoin, phenobarbital, and omeprazole) were excluded (27).

*Medication and blood sampling*

All the patients were treated with daily aspirin regimen with the dose of 80 to 325 for more than 1 week and 600 mg loading dose of clopidogrel (Plavix^®^; Sanofi Aventis, Bridgewater, New Jersey) at least 24 h before intervention. Medication continued after PCI with clopidogrel, 150 mg/d for 2 weeks and then 75 mg/d for 12 months, and aspirin 325 mg/d for 1 week and 80 mg/d for an indefinite period. The patients were followed up weekly by phone call until 1 month. Unfractionated heparin (50-70 IU/kg), as a bolus was administered to all patients in the catheterization laboratory immediately before stenting.

Blood samples were drawn in tube containing 3.8% Na-citrate at baseline (prior to procedure), 2 hours after receiving clopidogrel, 24 h and 30 days after stenting. To minimize environmental effects, laboratory tests (platelet aggregation and hematology assays) were performed within 2–3 hours after sampling.

*Platelet aggregation*

The blood-citrate mixture was centrifuged at 800 rpm for 8 min to recover platelet-rich plasma (PRP) and further subjected to centrifugation at 4000 rpm for 20 min to recover platelet-poor plasma (PPP). The PRP and PPP were stored at the room temperature to be used within 2 h. The platelet count was determined in the PRP sample and adjusted to 250 × 103/µL - 300 × 103/µL with PPP.

Platelet aggregation was assessed by stimulating PRP through the concentrations of 5 and 20 µL ADP (Helena BioSciences, Europe, Sunderland) on the basis of gold standard platelet function assessment; light transmittance aggregometry (LTA; Helena PACKS-4).

*Definition of clopidogrel responsiveness*

Responsiveness was defined as the relative platelet inhibition (RI) induced by the addition of clopidogrel, RI = [(pretreatment aggregation – post treatment aggregation)/(pretreatment aggregation) × 100. Clopidogrel responsiveness was classified into poor-responders and responders with RI ≤ 30% and > 30%, respectively (28).

*Genomic extraction and Genotyping*

Deoxyribonucleic acid (DNA) was isolated from peripheral blood lymphocytes with High yield DNA Purification kit (CinnaGen Inc., Tehran, Iran). For genotyping of C3435T single nucleotide polymorphisms in ABCB1 gene, restriction fragment length polymorphism-polymerase chain reaction (PCR-RFLP) assay was performed. The sense (5’-TGC TGG TCC TGA AGT TGA TCT GTC AAC-3’) and anti-sense (5’-ACA TTA GGC AGT GAC TCG ATG AAG GCA-3’) specific primers were used to generate 248 base pair fragments. PCR reaction mixture (50 µL) includes 0.28 µM of each primer, a buffer containing 10 µM Tris, 50 µM KCl, 1.5 µM MgCl2, 200 µM each of dNTP and 1 U Taq DNA polymerase. Briefly, the PCR amplification condition included 8 min of initial denaturation step at 94 °C, followed by 35 cycles of melting at 94 °C for 30 s, annealing at 68 °C for 30 s, and elongation at 72 °C for 30 s and a final extension step for 10 min at 72 °C. Subsequently, PCR products were digested at 37 °C overnight with restriction enzyme Mbo I (Roche, Germany) and inactivated at 65 °C for 20 min. Digested fragments were run on a 3% agarose gel. Genotypes were determined according to the banding pattern.

*Statistical analysis*

Continuous and categorical data were summarized as mean ± standard error and percentage, respectively. The patients were divided into the poor responder and responder based on the RI. The assessment of conformity with a normal distribution was done using the Kolmogorov-Smirnov test. Deviation from Hardy-Weinberg equilibrium of allele frequencies was performed by Arlequin software version 3.1 using chi-square test. Mann Whitney U test was used to compare RI between patients with or without T allele. The comparison of wild-types and variant subjects genotype frequencies and demographic characteristics between the responder and poor responder were done using Pearson Chi-Square and Fisher’ Exact tests. The *P*-value below 0.05 was considered as significant. Finally, the data assembled in SPSS 17 (SPSS Inc., Chicago, Illinois) and GraphPad Prism 5.0 (GraphPad, La Jolla, CA, USA) software packages.

## Results

*Patients and outcomes*

Sixty-seven patients participated in this study. Demographic characteristics, clinical information and lab data of patients are presented in the Tables 1 and 2 for both 5 and 20 µM of ADP, respectively. The comparison of demographic characteristics between the responder and poor-responder groups indicated no significant differences (*P* > 0.05).

Based on RI results, the poorest responsiveness of clopidogrel was 18.6% and 18.3% after 2 hours of administration of 600 mg clopidogrel, measured in concentration of 5 and 20 µM ADP, respectively. Percentage of the poor responder and the responder groups in each 5 and 20 µM at 2 and 24 h and also 30 days after stenting are shown in Table 3.

*ABCB1 genetic polymorphism*

**Figure 1:**
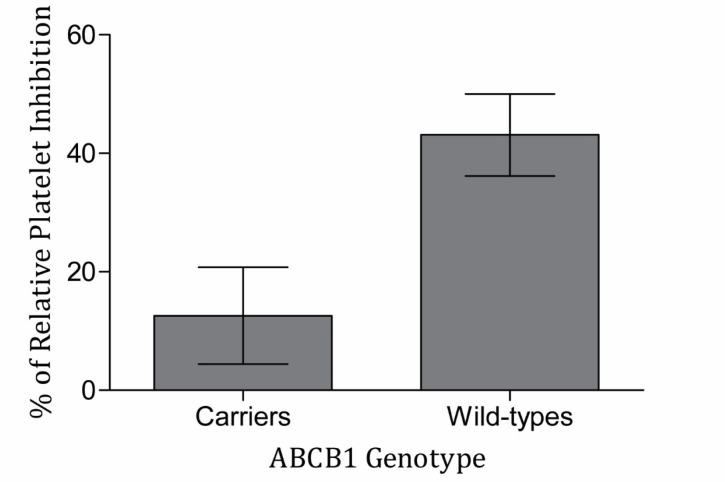
Relative platelet inhibition of clopidogrel responsiveness in T allele carriers and wild-type subjects (N = 66). Blood samples were collected 2 h after receiving clopidogrel loading dose 600 mg. Then, the platelet aggregation was measured with stimulating platelet-rich plasma by the concentration of 5 µM ADP with light transmittance aggregometry. Difference of relative platelet inhibition with 5 µM ADP was marginally significant between wild-type and T allele carriers in patients administrated by clopidogrel (*P* = 0.056). Data are presented as mean ± SEM of the relative platelet inhibition

Among the 67 patients enrolled in this study, 14 (20.9%), 50 (74.6%) and 3 (4.5%) patients had wild type (CC), heterozygote (CT) and TT genotypes, respectively. due to low frequency of TT genotype subjects, statistical analysis was done between wild type subjects and carriers of T allele. The allele frequencies were deviated from Hardy-Weinberg equilibrium. The comparison of genotype frequencies, wild-type subjects, and T allele carriers between responders and the poor responders revealed no significant differences at all time points by both 5 and 20 µM ADP. There was no significant difference of RI among wild-type and variant subjects in the ABCB1 genetic polymorphism in all circumstances. It is of note that the mean of RI in the wild-type subjects were higher than T allele carriers in both ADP concentrations and all time points, particularly following 2 h (Figure 1) (Table 4).

## Discussion

Clopidogrel is a well-studied anti-platelet agent (29, 30). However, inter-patients variability was remained as a though challenge in up to 30% of patients treated with clopidogrel (6). 

Several environmental, clinical and genetic factors involve in clopidogrel response variability (31-34). In this study, demographic characteristics including age, smoking, diabetes mellitus, lipid profile, and body mass index (BMI) were not associated with clopidogrel response variability (*P* > 0.05). The same finding has been reported about the association of the environmental and clinical factors and clopidogrel responsiveness in other populations (5, 6 and 35). Also, there are reports showing that some factors such as age, smoke, and diet may affect the inter-patients variability in response to clopidogrel in other populations (36, 37).

Several pharmacogenetic studies proposed the potential associations of clopidogrel heterogeneity response and genetic polymorphism of molecules that are involved in clopidogrel metabolism and absorption (9-12, 38). Our previous study on the Iranian population showed that P2Y12, CYP3A5 and CYP2C19 polymorphism were not responsible for clopidogrel inter-individual variability (17). Furthermore, we observed minimum RI induced by clopidogrel after 2 h of taking 600 mg loading dose clopidogrel. This led us to think about the possible disruption of clopidogrel absorption through the intestinal P-gp.

Based on RFLP-PCR results, 14 (29.9%) of patients had CC genotype and 50 (74.6%) and 3 (4.5%) of patients had CT and TT genotypes, respectively. The frequency of the T allele in the present study was 42%. This frequency is lower than previous study in Iranian healthy subjects with 54% frequency of T allele (39). T allele frequency in other populations was reported as follows, 52.4% of Indian (40), 21% of African (41), 42% of Asian and 55% of Caucasians (42).

The RI of clopidogrel did not reveal significant differences between T allele variant and wild-type subjects in both ADP concentrations and time points. However, we found a tendency in the increase of the RI by wild-types compared to variants. This might occur due to decreased P-gp activity in the wild-type patients, leading to higher absorption or blood concentration of clopidogrel. The effect of ABCB1 C3435T polymorphism on P-gp expression and activity has been investigated in the previous studies. Hoffmeyer *et al.* showed that volunteer homozygous for T allele had lower expression and activity (highest concentration of digoxin) of P-gp (43). Other parallel *in-vivo* studies showed that C3435T variant alleles are associated with increased P-gp activity (10, 44). Moreover, Hemauer *et al.* demonstrated that C3435T variant alleles are significantly associated with lower P-gp expression and speculated that this gene regulation can be secondary to a rise in P-gp transport activity of variant subjects (44).

Conflicting results of many studies have been also reported about the association between ABCB1 polymorphisms and clopidogrel responsiveness. A Chinese cohort has suggested there is no association between clopidogrel responsiveness and ABCB1 polymorphism. The T allele frequency in this population was 41%, very similar to our findings (45). A meta-analysis including 6 studies was also failed to find any association between ABCB1 polymorphisms and overall recurrent ischemic events, thrombosis or bleeding (46). On the other hand, findings of TRITON TIMI-38 trial revealed that C3435T homozygous TT had reduced platelet inhibition and this genotype is associated with increased cardiovascular death, myocardial infarction and stroke during treatment with clopidogrel. The genotype frequencies of TT, CT and CC in this trial were 27%, 50% and 23%, respectively (T allele frequency was 52%) (47). Similar frequencies were reported in 2208 patients with acute myocardial infarction receiving clopidogrel therapy (9). PLATO trial reported that CC genotypes in the clopidogrel group have higher ischemic events in comparison to CT or TT genotypes. 

One of the limitations of our study is lack of ABCB1 polymorphism assessment, influencing on blood clopidogrel concentration or its clinical outcomes. However, a recent study including 42 patients with coronary artery diseases showed that the plasma level of clopidogrel was significantly lower in TT genotype, while no association between platelet aggregation and ABCB1 polymorphisms was observed (52). Wang *et al.* found that T allele was associated with lower plasma clopidogrel level and its active metabolite, significantly which was also associated with consequently decreased platelet inhibition (*P* < 0.05) (53). Another limitation is the small sample size of wild type group. Due to small sample size, the study was under power and its generalizability is limited.

Several factors may be responsible for these contradictory data including the number of patients, combination of susceptibility variant, the nature of research population, heterogeneity of the diseases especially cardiovascular diseases, and gene-environmental interactions (54). Moreover, severity of genetic mutation on drug response could be attenuated or exaggerated by other genetic polymorphisms and environmental factors (55). Taking the latter into consideration, assessment of other SNPs such as G2677T/A could be of interest. 

Overall, in the present study, we did not find association between ABCB1 polymorphism (C3435T) and inter-individual RI variability of clopidogrel in Iranian patients undergoing percutaneous coronary intervention. ABCB1 polymorphism (C3435T) may not attribute to delayed action of the loading dose of clopidogrel in comparison to other populations.

**Table 1:** Demographic characteristics, clinical information, and lab data of patients (N = 67). No significant differences between responder and poor responder in patient's characteristics were observed at all times by 5 µM ADP (*P* > 0.05).

		**Total**	**ADP 5 µM**					
			**2 h**		**24 h**		**30 days**	
			**P** ^f^	**R** ^g^	**P**	**R**	**P**	**R**
Age (year), mean ± SD		57.78 ± 10.80	57.03 ± 10.29	58.70 ± 11.38	56.47 ± 9.50	58.67 ± 11.57	56.23 ± 9.64	58.88 ± 11.56
Sez	47 (70.1)	23 (50.0)	23 (50.0)	15 (33.3)	30 (66.7)	16 (35.6)	29 (64.4)	29 (64.4)
	20 (29.9)	9 (45.0)	11 (55.0)	4 (21.1)	15 (78.9)	5 (26.3)	14 (73.7)	14 (73.7)
Smoking, NO (%)		27 (40.3)	13 (48.1)	14 (51.9)	8 (29.6)	19 (70.4)	9 (33.3)	18 (66.7)
Diabetes Mellitus, NO (%)		14 (20.9)	7 (50.0)	7 (50.0)	5 (35.7)	9 (64.3)	5 (35.7)	9 (64.3)
HTN^a^ (BP ≥140/90 mmHg), NO (%)		32 (47)	13 (41.9)	18 (58.1)	5 (16.1)	26 (83.6)	9 (29.0)	22 (71.0)
Hyperlipidemia (LDL-C≥100 mg/dL), NO (%)		49 (73.1)	22 (45.8)	26 (54.2)	14 (29.8)	33 (70.2)	16 (34.0)	31 (66.0)
BMI^b^ (**k**g/m^2^), mean ± SD		26.25 ± 4.02	27.40 ± 4.23	25.09 ± 3.54	27.21 ± 4.02	25.89 ± 4.03	27.56 ± 4.71	25.68 ± 3.62
LVEF^c^<45%, NO (%)		6 (9.0)	3 (50.0)	3 (50.0)	2 (33.3)	4 (66.7)	2 (33.3)	4 (66.7)
WBC^d^ (× 1000/µL), mean ± SD		7.28 ± 2.16	6.95 ± 1.63	7.64 ± 2.56	7.75 ± 2.87	7.04 ± 1.81	7.14 ± 1.92	7.31 ± 2.32
PLT^e^ (× 1000/µL), mean ± SD		229.9 ± 67.48	219.44 ± 59.71	241.82 ± 73.24	226.37 ± 65.81	226.37 ± 65.81	225.19 ± 58.97	234.07 ± 73.21

A: hypertension; b: body mass index; c: left ventricular ejection fraction; d: white blood cell; e: platelet; f: poor responder; g: responder.

**Table 2:** Demographic characteristics, clinical information, and lab data of patients (N = 67). No significant differences between responder and poor responder in patient's characteristics were observed at all times by 20 µM ADP (*P* > 0.05).

		**Total**	**ADP 20 µM**					
			**2 h**		**24 h**		**30 days**	
			**P** ^f^	**R** ^g^	**P**	**R**	**P**	**R**
Age (year), mean ± SD		57.78 ± 10.80	58.22 ± 9.65	57.39 ± 11.79	57.46 ± 10.26	58.00 ± 11.17	56.87 ± 8.31	58.20 ± 11.65
Sez	47 (70.1)	22 (46.8)	25 (53.2)	10 (21.7)	36 (78.3)	12 (26.1)	34 (73.9)	29 (64.4)
	20 (29.9)	9 (45.0)	11 (55.0)	3 (15.8)	16 (84.2)	3 (15.8)	16 (84.2)	14 (73.7)
Smoking, NO (%)		27 (40.3)	12 (44.4)	15 (55.6)	5 (18.5)	22 (81.5)	5 (18.5)	22 (81.5)
Diabetes Mellitus, NO (%)		14 (20.9)	6 (42.9)	8 (57.1)	3 (21.4)	11 (78.6)	2 (14.3)	12 (85.7)
HTN^a^ (BP ≥140/90 mmHg), NO (%)		32 (47)	16 (50.0)	16 (50.0)	6 (18.8)	26 (81.2)	9 (28.1)	23 (71.9)
Hyperlipidemia (LDL-C≥100 mg/dL), NO (%)		49 (73.1)	20 (40.8)	29 (59.2)	10 (20.8)	38 (79.2)	13 (27.1)	35 (72.9)
BMI^b^ (kg/m^2^), mean ± SD		26.25 ± 4.02	27.48 ± 4.50	25.26 ± 4.34	26.45 ± 3.91	25.25 ± 3.34	27.48 ± 4.20	26.07 ± 3.99
LVEF^c^<45%, NO (%)		6 (9.0)	1 (16.7)	5 (83.3)	0 (0.0)	6 (100.0)	0 (0.0)	6 (100.0)
WBC^d^ (× 1000/µL), mean ± SD		7.28 ± 2.16	7.12 ±1.70	7.42 ± 2.51	8.14 ± 3.29	7.00 ± 1.76	8.31 ± 2.87	6.90 ± 1.82
PLT^e^ (× 1000/µL), mean ± SD		229.9 ± 67.48	219.42 ± 60.54	239.00 ± 72.54	232.69 ± 66.35	229.44 ± 69.66	234.80 ± 62.03	228.68 ± 70.88

A: hypertension, b: body mass index, c: left ventricular ejection fraction, d: white blood cell, e: platelet, f: poor responder, g: responder

**Table 3:** Percentage of patients in the responder or the poor responder groups at different ADP concentrations and time points (N = 67).

**Percent of Miss data**	**Percent of Poor responder**	**Percent of Responder**	**ADP concentration-time of blood sampling**
1.5	47.8	50.7	5^a^-2^b^
4.5	28.4	67.2	5^a^-24^b^
4.5	31.3	64.2	5^a^-30^c^
0	46.3	53.7	20^a^-2^b^
3	19.4	77.6	20^a^-24^b^
3	22.4	74.6	20^a^-30^c^

^a^µM; ^b^hours after stenting; ^c^Day after stenting

**Table 4:** Comparison of relative platelet inhibition (RI) means between T allele carriers (Carrier) and wild type (CC) of ABCB1 polymorphisms at different time points of clopidogrel administration and concentrations of ADP (N = 67).

**Time of RI after clopidogrel loading dose**	**ADP conc.** **(µM)**	**genotype**	**Number**	**Mean ± Standard error of RI**	***p*** **-value**
2 h	5	Carrier	53	12.60 ± 8.16	0.056
		CC	13	43.11 ± 6.92	
24 h	5	Carrier	52	37.02 ± 8.00	0.082
		CC	12	62.36 ± 8.65	
30 d	5	Carrier	52	35.98 ± 9.06	0.918
		CC	12	41.78 ± 10.06	
2 h	20	Carrier	54	14.83 ± 8.55	0.419
		CC	13	32.74 ± 7.53	
24 h	20	Carrier	53	39.10 ± 10.20	0.116
		CC	12	59.79 ± 8.93	
30 d	20	Carrier	53	38.38 ± 11.62	0.588
		CC	12	40.05 ± 10.61	
